# Health outcomes in primary care: a 20-year evidence map of randomized controlled trials

**DOI:** 10.1093/fampra/cmac067

**Published:** 2022-07-09

**Authors:** Aristea Missiou, Christos Lionis, Evangelos Evangelou, Athina Tatsioni

**Affiliations:** Research Unit for General Medicine and Primary Health Care, Faculty of Medicine, School of Health Sciences, University of Ioannina, Ioannina, Greece; Clinic of Social and Family Medicine, School of Medicine, University of Crete, Crete, Greece; Department of Health, Medicine and Care, General Practice, Linköping University, Linköping, Sweden; Department of Hygiene and Epidemiology, Faculty of Medicine, School of Health Sciences, University of Ioannina, Ioannina, Greece; Department of Epidemiology and Biostatistics, Imperial College London, London, United Kingdom; Research Unit for General Medicine and Primary Health Care, Faculty of Medicine, School of Health Sciences, University of Ioannina, Ioannina, Greece

**Keywords:** evidence-based practice, outcome assessment, primary health care, quality of health care, randomized controlled trial, systematic review

## Abstract

**Objective:**

To quantify the different types of health outcomes assessed as primary outcomes in randomized controlled trials (RCTs) in the primary care (PC) setting during the last 20 years and identify whether potential gaps exist in specific types of health care and types of intervention.

**Methods:**

We systematically searched PubMed, Scopus, and Cochrane Central Register of Controlled Trials, from January 2000 to September 2020 for published RCTs in PC. We recorded characteristics of eligible studies and mapped evidence by health outcome category (patient health outcomes, health services outcomes); and for each outcome category, by types of health care (preventive, acute, chronic, palliative), and by types of intervention (drug, behavioural, on structure, and on process). For RCTs assessing patient health outcomes as primary outcomes, we further mapped using the quality-of-care dimensions, that is, effectiveness, safety, and patient-centredness.

**Results:**

Of the 518 eligible RCTs in PC, 357 (68.9%) evaluated a patient health outcome as the primary outcome, and 161 (31.1%) evaluated only health services outcomes as primary outcomes. Many focused on population with chronic illness (224 trials; 43.2%) and evaluated interventions on processes of health care (239 trials; 46.1%). Research gaps identified include preventive and palliative care, behavioural interventions, and safety and patient-centredness outcomes as primary outcomes.

**Conclusion:**

Our evidence map showed research gaps in certain types of health care and interventions. It also showed research gaps in assessing safety and measures to place patient at the centre of health care delivery as primary outcomes.

**Key messages**

- RCTs in primary care assessed mainly patient health outcomes in chronic care.
- Few trials looked at preventive care; even fewer trials looked at palliative care.
- RCTs mainly evaluated drugs and interventions on processes of care.
- A limited number of trials evaluated behavioural interventions.
- Few trials assessed safety and patient-centredness as primary outcomes.

## Background

According to the Organization for Economic Co-operation and Development (OECD) framework, quality in health care means that provided care is effective, safe, and patient-centred.^1,2^ A strong primary care (PC) system is considered the cornerstone in health systems for delivering high-quality care.^3^ PC, fundamental to medical practice, entails the provision of individual patient care; the broader primary health care (PHC) entails population-level public health-type services.^4,5^ The response to the coronavirus disease 2019 (COVID-19) pandemic has demonstrated the key role of PC from planning, surveillance, to prevention (e.g. masking, vaccination) along with the need to ensure patient safety and maintain provision of essential health services.^6^

Research constantly indicates robust association between PC and lower costs, better utilization patterns, and reduced mortality.^3^ Previous studies have shown that differences in PC support could partly explain health outcome differences across developed countries.^7^ Therefore, PC models should be evaluated and disseminated not only on achieving short-term cost savings but also on their ability to deliver high-quality care.^3^ The identification of effective and safe interventions that are responsive to patients’ needs, and the recognition of potential knowledge gaps, will inform evidence-based decision-making on future research priorities and policy making.

Previous papers have commented on the fair to poor quality of PC research and the necessity for a specific research agenda.^8–10^ Despite previous extended efforts, there is still difficulty in interpreting the results of PC studies reporting either original research or systematic reviews.^11^ The reported lack of proven causalities between PC characteristics, such as continuity, integration, and coordination, and study outcome measures has been suggested as a contributing factor.^11^ Another reason may be the small number of randomized controlled trials (RCTs) with robust evidence in PC.^12^ Moreover, systematic review authors often do not comment on the applicability of findings in the PC field even though they have included PC studies.^13^ Uncertainty in harm-benefit ratio for PC interventions due to inadequate robust evidence may differ between patient health outcomes and health services outcomes. Patient health outcomes include outcome measures indicating the impact of the intervention on patient health status (e.g. cardiovascular event, quality of life); health services outcomes include population outcomes (e.g. average life expectancy), system outcomes (e.g. readmission rate, timeliness of care), costs, and others.^14^ A systematic effort to identify RCTs in PC that have assessed these two outcome categories, and potential gaps that still exist is yet to be taken.

To address this issue, we mapped the evidence in the field of PC.^15,16^ Evidence mapping may provide a comprehensive summary of the extent and distribution of the evidence in a broad clinical area, allowing a snapshot of where evidence exists and where it is lacking.^17^ Our study aimed at systematically identifying published RCTs in PC, mapping them according to their primary outcome category, and unveiling potential gaps for future research in specific types of health care (preventive, acute, chronic, and palliative) and types of intervention (drug, behavioural, on structure, and on the process of care).

## Methods

We reported this study according to Preferred Reporting Items for Systematic Reviews and Meta-Analyses extension for Scoping Reviews (PRISMA-ScR) guidelines (Supplementary Table S1).^18^

### Search strategy

We searched PubMed, Scopus, and Cochrane Central Register of Controlled Trials (CENTRAL) from January 2000 to September 2020 for published RCTs in PC. For PubMed, we used a search strategy including keywords related to family medicine/general practice, and primary care, combined with the Cochrane Collaboration search algorithm for RCTs. We conducted a systematic search on Scopus using the same keywords after excluding articles registered in MEDLINE. We excluded items manually in Scopus that were published in journals indexed in PubMed since they had already been screened in the PubMed search. Finally, we searched CENTRAL. After excluding articles that were registered either in PubMed or Scopus, we screened the remaining items. Search algorithms were described in detail in Supplementary Table S2.

Two investigators (AM and AT) independently performed a pilot screening based on the title and/or abstract for the first 500 items in PubMed to identify potential disagreements in the screening process; the screening process was finalized after these disagreements were resolved. One investigator (AM) completed the screening of all titles and/or abstracts in PubMed, Scopus, and CENTRAL. For items considered potentially eligible or unclear, the full text was retrieved. A second investigator (AT) checked on items that the first investigator (AM) could not decide. Discrepancies were resolved through consensus. For trials where the full text could not be retrieved or insufficient information was provided to render an eligibility decision, the investigators were contacted when an e-mail address was available. Two consecutive reminders were sent to non-responders.

### Eligibility criteria

Only RCTs published since 2000 in the English language involving human participants were considered. We included all eligible RCTs regardless of their quality. We included RCTs only if the authors clearly stated that their trial was carried out exclusively in a PC setting. PC concerns the narrower concept of family physician-type services delivered to individuals without entailing public health functions, such as community health services, and health promotion programmes.^4,5^ More specifically, an RCT was considered relevant to PC if investigators clarified that they had recruited participants from PC, or general practice, or family medicine and that a PC professional, or the PC team, had been involved in the intervention. We considered eligible interventions that were applied individually to the participants. We expected substantial diversity of professionals included in a PC team among different countries, and heterogeneity regarding their skills, training, and field of expertise. For example, health professionals, such as dieticians, pharmacists, lay health workers, etc. are part of the PC team in some countries, while in other countries they are not. Regardless, to be eligible, the study must include a core PC team including the following participating professionals: PC physicians, general practitioners/family medicine physicians, nurses (without specialized training), and medical assistants.

The following types of studies were excluded: (a) Studies with a mixed population, that is, PC patients and patients from specialized outpatient clinics, a mixed intervention, that is, an intervention involving a health professional outside the PC team as defined by eligibility criteria, such as physiotherapy or psychotherapy in PC, or mixed settings, that is, PC practice and hospital outpatient clinic providing specialized care; (b) Studies that evaluated large-scale public health promoting interventions, reimbursement and/or monetary incentives; and studies on complementary medicine; (c) Trials that evaluated outcomes on health care professionals, that is, change in knowledge, attitude, and behaviour but did not evaluate any outcomes on patients, or health services outcomes; (d) Pilot or feasibility studies, published protocols, economic analyses, abstracts or conference proceedings, and dissertations or theses; (e) Studies involving only follow-up analyses after completion of the study; studies reporting only subgroup or post hoc analyses; and studies reporting a mid-term interim analysis, or preliminary result analyses, or baseline results without final endpoint results.

### Data extraction

For each eligible RCT, the following were determined: whether the primary outcome was (a) a patient health outcome, or (b) a health service outcome according to Health Care Quality Measures classification as described by Agency for Healthcare Research and Quality (AHRQ).^14^ We considered patient health outcome as any outcome measure reflecting the impact of the intervention on the health status of patients.^14^ Patient health outcomes may be either clinician-, observer-, or self-reported [patient-reported outcome measures (PROMs)] or performance-based.^19^ Specific examples of patient health outcomes are control of blood pressure (BP), level of haemoglobin A1c, the score on a patient self-rating depression severity scale, and others. Health services outcomes are any measure that reflects the effect of an intervention on the structure (e.g. wraparound service) or process of health care or care delivery (e.g. population-based screening programme). Specific examples of health services outcomes include the percentage of people having their BP measured in a PC encounter, the proportion of patients at risk for diabetes being screened, the depression detection rate among patients being at risk, and others.

We further categorized patient health outcomes according to the quality-of-care dimension described by OECD Health Care Quality Framework, that is, outcomes on effectiveness, safety, and patient-centredness.^2^ For studies evaluating more than one primary outcome, we captured them all and categorized them accordingly. We merged studies that evaluated both patient health and health services outcomes as primary outcomes, with studies that evaluated only patient health outcomes. Thus, we compared RCTs assessing at least one patient health outcome as primary outcome with RCTs assessing only health services outcomes as primary outcomes.

We also recorded characteristics, such as publication year, geographical region according to United Nations Statistics Division, funding source category [government or public, non-governmental organization or institute, mixed (industry not included), mixed including industry, industry, and no funding], and whether it was a cluster RCT or not. We further extracted the unit of randomization (setting, health care professional, and patient); the type of health care according to the OECD framework, that is, preventive care (staying healthy), acute care (getting better), chronic care (living with illness or disability), palliative care (coping with end of life)^2^; the type of intervention, that is, drug, food supplement, or device, behavioural intervention (on patients), intervention on the structure (e.g. training programme for health professionals on motivational interviewing), intervention on processes of care (e.g. use of a decision aid); and whether the intervention was provided to health care professionals, for example, a training skill programme for professionals, and multidisciplinary communication facilitators; or whether it was provided to patients.

### Statistical analysis

Data were presented as absolute numbers and frequencies for binary and categorical variables, and as median with interquartile range (IQR) for continuous variables. We presented separately characteristics for trials assessing at least one patient health outcome, and for studies assessing only health services outcomes as the primary outcome. Comparisons were performed using the Pearson’s chi-square test or Fisher’s exact test for discrete variables and the Mann–Whitney test or Kruskal–Wallis test for continuous variables as appropriate. To visualize research gaps, we created heatmaps, grouping studies by outcome categories, types of health care, and types of intervention. For all comparisons, we considered statistically significant an adjusted *P*-value threshold of less than 0.003 after applying Bonferroni correction for multiple comparisons. Unadjusted *P*-values were reported throughout the text and tables. All analyses were performed using IBM SPSS Statistics for Windows, Version 26.0 (IBM Corp., Armonk, NY, USA) and Microsoft Excel, MS Office 2019 (Microsoft Corp., Redmond, WA, USA).

## Results

### Eligible studies

**Fig. 1.**
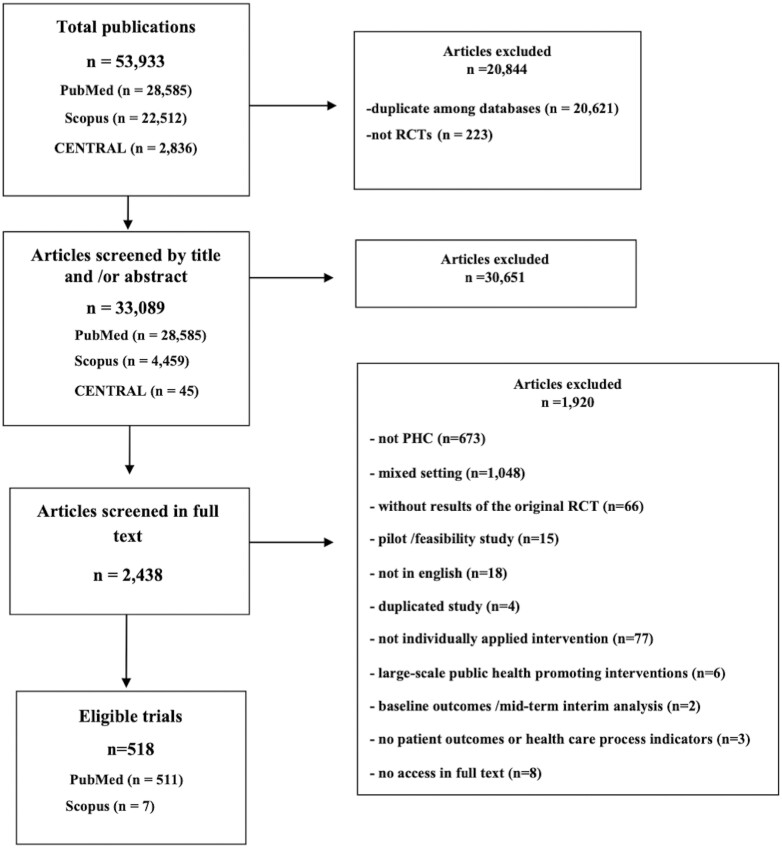
Flowchart of eligible studies published during 2000–2020.

Our search yielded a total of 53,933 items between January 2000 and September 2020. After applying the eligibility criteria, 518 articles were considered eligible; 511 were found in PubMed, and 7 in Scopus (Fig. 1).

### Characteristics of eligible studies

**Fig. 2.**
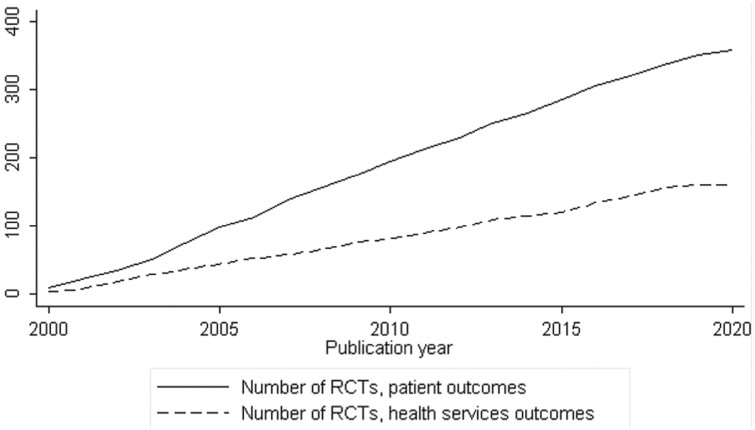
Total number of published RCTs per primary outcome category over time (2000–2020).

The total number of published RCTs per outcome category over time is increasing (Fig. 2). Out of the 518 eligible RCTs, 357 (68.9%) evaluated a patient health outcome as the primary outcome, and 161 (31.1%) evaluated only health services outcomes. There was no significant difference in median publication year (*P* = 0.56), and in geographic region distribution (*P* = 0.52) for RCTs between outcome categories. Supplementary Figure S1 shows the geographical distribution of PC RCTs at the country level.

Table 1 presents the characteristics of eligible studies per primary outcome category. Most RCTs were conducted in Europe in both outcome categories. RCTs assessing patient health outcomes, generally included patients as the randomization unit (256 trials; 71.7%), evaluated interventions that were usually applied on patients (268 trials; 75.1%), and were mainly funded by sponsors that did not include industry (196 trials; 54.9%), whereas industry was the funder in about one-third of the cases (*P* < 0.001 between outcome categories). RCTs evaluating health services outcomes often had a cluster design (122 trials; 75.8%), generally included the setting as the unit of randomization (105 trials; 65.2%), evaluated interventions that were usually applied on health professionals (104 trials; 64.6%) and were rarely funded by industry (*P* < 0.001 between outcome categories).

**Table 1.** Characteristics of the 518 eligible studies per primary outcome category published during 2000–2020.

Characteristic	RCTs assessing patient health outcomes, *N* = 357	RCTs assessing health services outcomes, *N* = 161	Unadjusted *P*-value^a^
Country region			
Europe, *n* (%)	221 (61.9)	103 (64.0)	0.52
America, *n* (%)	80 (22.4)	40 (24.8)	
Oceania, *n* (%)	21 (5.9)	9 (5.6)	
Asia, *n* (%)	22 (6.2)	5 (3.1)	
Africa, *n* (%)	8 (2.2)	4 (2.5)	
Multiple regions, *n* (%)^b^	5 (1.4)	0	
Study design			
Cluster RCT, *n* (%)	105 (29.4)	122 (75.8)	<0.001
Unit of randomization			
Setting, *n* (%)	53 (14.8)	105 (65.2)	<0.001
Health professional, *n* (%)	48 (13.4)	32 (19.9)	
Patient, *n* (%)	256 (71.7)	24 (14.9)	
Intervention unit			
Health professionals, *n* (%)	26 (7.3)	104 (64.6)	<0.001
Patients, *n* (%)	268 (75.1)	28 (17.4)	
Both health professionals and patients, *n* (%)	63 (17.6)	29 (18.0)	
Funding source type			
Government/public, *n* (%)	127 (35.6)	77 (47.8)	<0.001
Non-governmental organization/institute, *n* (%)	43 (12.0)	16 (9.9)	
Mixed (industry not included), *n* (%)	26 (7.3)	21 (13.0)	
Mixed (industry included), *n* (%)	29 (8.1)	12 (7.5)	
Industry, *n* (%)	95 (26.6)	14 (8.7)	
Not reported, *n* (%)	27 (7.6)	16 (9.9)	
No funding, *n* (%)	10 (2.8)	5 (3.1)	

We considered statistically significant an adjusted threshold of *P* < 0.003, after applying Bonferroni correction for multiple comparisons.

Multiple regions: Europe and America (*n* = 2); Europe and Oceania (*n* = 1); Asia and Africa (*n* = 1); Europe, America, and Africa (*n* = 1).

### Types of health care and types of interventions evaluated in PC RCTs

Table 2 shows the types of health care and the types of interventions that were evaluated in PC RCTs. RCTs more often focused on chronic and acute care needs, less often on preventive care, and rarely on palliative care needs. The two trials (0.6%) on palliative care assessed patient health outcomes as primary outcomes. Twelve trials (7.5%) addressing multiple health care types evaluated only health services outcomes as primary outcomes [*P* < 0.001 between outcome categories]. RCTs assessing patient health outcomes as primary outcomes, evaluated a drug, a food supplement, or a device (142 trials; 39.8%), and interventions on processes of care (129 trials; 36.1%). RCTs assessing only health services outcomes as primary outcomes, evaluated interventions on structure (51 trials; 31.7%), and on processes of care (110 trials; 68.3%) [*P* < 0.001 between outcome categories].

**Table 2.** Type of health care and type of interventions per primary outcome category in 518 RCTs conducted in primary care during 2000–2020.

Health care type/intervention type	Patient health outcomes, *N* = 357	Health services outcomes, *N* = 161	Unadjusted *P*-value^a^
Health care type			
Preventive care, *n* (%)	77 (21.6)	38 (23.6)	0.001
Acute care, *n* (%)	113 (31.7)	49 (30.4)	
Chronic care, *n* (%)	162 (45.4)	62 (38.5)	
Palliative care, *n* (%)	2 (0.6)	0	
Multiple health care types, *n* (%)^b^	3 (0.8)	12 (7.5)	
Type of intervention			
Drug/food/device, *n* (%)	142 (39.8)	0	<0.001
Behavioural intervention (on patients), *n* (%)	47 (13.2)	0	
Intervention on structure, *n* (%)	36 (10.1)	51 (31.7)	
Intervention on processes of care, *n* (%)	129 (36.1)	110 (68.3)	
Multiple types of intervention, *n* (%)^c^	3 (0.8)	0	

We considered statistically significant an adjusted threshold of *P* < 0.003, after applying Bonferroni correction for multiple comparisons.

Multiple health care types: RCTs with patient health outcomes: chronic and acute care (*n* = 1); chronic and preventive care (*n* = 2), RCTs with health services outcomes: chronic and acute care (*n* = 7); chronic and preventive care (*n* = 3); chronic, acute, and preventive care (*n* = 2).

Multiple types of intervention: behavioural intervention (on patients) and intervention on structure (*n* = 1); behavioural intervention (on patients) and a drug (*n* = 2).

### Quality-of-care dimensions assessed in PC RCTs

Among the 357 RCTs assessing patient health outcomes as primary outcomes, the majority (254 trials, 71.1%) assessed the effectiveness of interventions as the primary outcome, 14 trials (3.9%) assessed safety as the primary outcome, and 67 trials (18.8%) patient-centredness as the primary outcome. In addition, 22 (6.2%) assessed both effectiveness and patient-centredness as primary outcomes.

Table 3 presents the associations of the type of health care and the type of the evaluated intervention with the quality-of-care dimension of the assessed patient health outcome. Generally, RCTs assessing effectiveness as the primary outcome addressed acute (90 trials; 35.4%) and chronic care needs (110 trials; 43.3%); RCTs assessing safety as the primary outcome addressed preventive (4 trials; 28.6%) and chronic care needs (9 trials; 64.3%); trials assessing patient-centredness as the primary outcome addressed preventive (17 trials; 25.4%), acute (16 trials; 23.9%), and chronic care needs (29 trials; 43.3%); RCTs assessing multiple quality dimensions addressed chronic care needs (14 trials; 63.6%) [*P* = 0.004 among quality-of-care dimension categories]; RCTs assessing effectiveness as the primary outcome evaluated a drug, a food supplement, or a medical device (131 trials; 51.6%), and interventions on processes of care (70 trials; 27.6%); RCTs assessing safety as the primary outcome, evaluated a drug, a food supplement, or a medical device (4 trials; 28.6%), interventions on structure (4 trials; 28.6%), and interventions on processes of care (5 trials; 35.7%); RCTs assessing patient-centredness as the primary outcome evaluated interventions on processes of care (44 trials; 65.7%); and RCTs assessing multiple quality dimensions evaluated interventions on processes of care (10 trials; 45.5%) [*P* < 0.001 among quality-of-care dimension categories] (Table 3).

**Table 3.** Type of health care and type of interventions per quality-of-care dimension (for 357 RCTs assessing patient health outcomes as primary outcomes, published during 2000–2020).

Health care type/intervention type	Quality-of-care dimension				Unadjusted *P*-value^a^
	Effectiveness, *N* = 254	Safety, *N* = 14	Patient-centredness, *N* = 67	Multiple quality dimensions^d^, *N* = 22	
Health care type					
Preventive care, *n* (%)	54 (21.3)	4 (28.6)	17 (25.4)	2 (9.1)	0.004
Acute care, *n* (%)	90 (35.4)	1 (7.1)	16 (23.9)	6 (27.3)	
Chronic care, *n* (%)	110 (43.3)	9 (64.3)	29 (43.3)	14 (63.6)	
Palliative care, *n* (%)	0	0	2 (3.0)	0	
Multiple health care types, *n* (%)^b^	0	0	3 (4.5)	0	
Type of intervention					
Drug/food/device, *n* (%)	131 (51.6)	4 (28.6)	4 (6.0)	3 (13.6)	<0.001
Behavioural intervention (on patients), *n* (%)	34 (13.4)	1 (7.1)	8 (11.9)	4 (18.2)	
Intervention on structure, *n* (%)	16 (6.3)	4 (28.6)	11 (16.4)	5 (22.7)	
Intervention on processes of care, *n* (%)	70 (27.6)	5 (35.7)	44 (65.7)	10 (45.5)	
Multiple types of intervention, *n* (%)^c^	3 (1.2)	0	0	0	

We considered statistically significant an adjusted threshold of *P* < 0.003, after applying Bonferroni correction for multiple comparisons.

Multiple health care types: chronic and acute care (*n* = 1); chronic and preventive care (*n* = 2).

Multiple types of intervention: behavioural intervention (on patients) and intervention on structure (*n* = 1); behavioural intervention (on patients) and a drug (*n* = 2).

Multiple quality-of-care dimensions: effectiveness and patient-centredness (*n* = 22).

### Using heatmaps to identify research gaps

Supplementary Figure S2 shows the heatmaps of RCTs per outcome category by type of health care and by type of intervention. Preventive care had generally few RCTs in all types of interventions except for behavioural interventions and interventions on processes of care among trials including patient health outcomes (*P* < 0.001) (Supplementary Fig. S2a). The number of trials in palliative care was sparse except for the few with interventions aiming at modifying processes of care (*P* < 0.001) (Supplementary Fig. S2a). Among trials assessing only health services outcomes as primary outcomes, there were no studies evaluating drugs, food supplements, devices, or behavioural interventions (*P* = 0.49) (Supplementary Fig. S2b). No eligible trials in palliative care were identified (*P* = 0.49) (Supplementary Fig. S2b).

Supplementary Figure S3 shows the heatmaps for RCTs including patient health outcomes per quality-of-care dimension and by type of intervention and type of health care. Trials on effectiveness as the primary outcome were scarce for interventions on structure, and none were identified that examined all types of interventions in palliative care (*P* < 0.001) (Supplementary Fig. S3a). There was a scarcity of RCTs reporting on safety as the primary outcome among all subgroups (*P* = 0.09) (Supplementary Fig. S3b). RCTs on patient-centredness as the primary outcome were also rare for all types of interventions and for all types of health care except for interventions on processes of care (*P* = 0.79) (Supplementary Fig. S3c). RCTs assessing both effectiveness and patient-centredness as primary outcomes were scarce for preventive care; and no studies were identified that evaluated drugs, food supplements, or devices in preventive care. No studies were identified that evaluated behavioural interventions in the acute care (*P* = 0.31) (Supplementary Fig. S3d).

## Discussion

Our study showed an increasing number of RCTs in PC, most of which were conducted in Europe and assessed patient health outcomes as primary outcomes. Cluster RCTs and interventions applied on health care professionals were predominant among trials assessing only health services outcomes as primary outcomes. Most trials evaluated interventions in chronic care. Interventions in preventive care were evaluated in almost one-fourth of the trials, while palliative care interventions were in short-supply. Most trials with patient health outcomes as primary outcomes evaluated drugs, food supplements, or devices, while behavioural interventions were scarce. All RCTs assessing only health services outcomes as primary outcomes evaluated interventions on structure and on processes of care. There were significant research gaps in trials assessing safety and patient-centredness as primary outcomes.

### Comparison with the literature

The scientific growth in PC still lags behind the number of RCTs in other medical disciplines.^20^ Recent articles on the COVID-19 pandemic also supported a scarcity of RCTs in PC.^21^ PC clinical practice guidelines are often supported from evidence extrapolated by other specialty fields, often of uncertain relevance to PC patients.^22^ Unlike other clinical fields, PC is based on a long-term relationship with the patients, and PC professionals have to factor in the family and other contextual factors including increasing social isolation, immigration, unemployment, and changes in the environment, on illness and health.^23^ Information from rigorously conducted RCTs are needed to guide the best care for a patient. However, we do acknowledge that for some types of interventions, RCTs may not be readily feasible because of cost, long time-horizon required, difficulty in enrolment, and other reasons. This may particularly apply to large-scale organizational interventions introduced as national policy. Yet, this problem is not unique to PC interventions and applies just as much to public health, social care, and secondary care interventions.

Most RCTs were conducted in Europe. This is in line with a previous report on published PC RCT protocols.^24^ Building PC research capacity for conducting RCTs in different regions could inform broadly applicable guidance. Cluster RCTs in PC may be a more pragmatic design to evaluate complex interventions,^25^ including interventions on health professionals that modify structures, and processes of care. Such interventions may incorporate main PC characteristics, including the patient’s first contact with the health care system, continuity, integration, and coordination of services.^26,27^ Specific outcomes that serve as health care indicators^18,28^ may guide recommendations on care delivery to improve health system performance. Complex interventions may act at multiple levels, incorporating features aiming both directly at patients and indirectly, through professionals and services, and vice versa. For example, they might aim at improved training for clinicians, so that they can provide more patient-centred consultations for patients; other interventions that aimed at patients will require some change in the behaviour or decisions of clinicians, and these changes in professional behaviour, in turn, will also require some form of intervention, for example, new guidance, protocols or training.

There were very few RCTs in preventive care. However, the identification of effective and safe PC interventions in preventive care may decrease premature mortality.^29–34^ A paucity in palliative care trials may partly reflect the difficulty of conducting such trials; however, palliative care interventions may support the identification of patients in need of end-of-life care, enhance the quality of life, and mitigate suffering among patients with a life-threatening illness.^35–38^ Interventions on PC structures and processes may also address inequalities in delivering preventive and palliative care.^39–42^ Besides drugs, future trials should identify appropriate behavioural interventions to reduce major risk factors, that is, sedentary lifestyle, diet, alcohol consumption, smoking, sexual behaviour, and violence.^43–45^ Behavioural interventions may change human behaviour, support self-care programmes, enhance long-term adherence, and improve health care indicators. Additionally, interventions on structures and processes may improve access in effective behavioural interventions limited by multiple barriers, including inadequate care delivery, workforce shortages, lack of outcome measurement, and payment methodologies that do not encourage high-value care.^46,47^

An explanation for under-reporting of safety and patient-centredness as primary outcomes might be that most RCTs evaluated drug interventions. However, trials that are not adequately powered to assess safety may pose substantial difficulties in assessing the net benefit.^48,49^ Inadequate reporting of side effects/adverse events was also recorded for other clinical fields.^50^ However, to facilitate researchers, funders, and policymakers, future RCTs need to assess safety outcomes in PC rigorously. To develop guideline recommendations and evidence-informed policy, all stakeholders need to be aware of the net benefit of interventions; thus, reporting safety issues are of paramount importance. In addition, adequately validated PROMs to measure patient-centredness were generally under-reported in health literature.^51^ For RCTs evaluating complex interventions, the outcome which is designated “primary” may be fairly notional, and often driven by a need to satisfy a funding body, which tends to prefer concrete health outcomes over “softer” outcomes such as patient-centredness. Thus, another explanation for recording a small number of specific types of outcomes may be because outcomes, such as patient-centredness, patient satisfaction, costs, changes in processes and use of health services, etc. have been collected as secondary outcomes, and therefore, will not have been identified in this review. The scarcity of PC RCTs focusing on safety and patient-centredness as primary outcomes may undermine the provision of high-value care.^52^

### Strengths and limitations

To the best of our knowledge, this is the first systematic effort to map PC RCTs of the last two decades to reveal potential research gaps. However, our study had several limitations. We included only RCTs published in English. Investigators working in a non-English-speaking country, that is, in South America, and Asia regions, may have published some of their work in local journals. Thus, we may have failed to capture non-English RCTs. That may partially explain a high proportion of studies from Europe published in English-language journals. We focused only on RCTs in PC; therefore, our results may not be generalizable for other study designs, and for community-oriented and public health trials. Excluding a broader PC team, as well as interventions in transition care, and in mixed care, our results may not be generalizable for multidisciplinary interventions, that is, collaborative, integrated, and rehabilitative care as included in PC according to WONCA Europe.^53^ This may also have yielded a limited number of trials from regions that provide PC care through a broader PC team or multidisciplinary health care models. To facilitate the interpretation of our results, we categorized interventions separately on structure, and on processes of care; however, we should consider that structure and process are inextricably linked in continuous interaction.^54^ We only captured what the authors mentioned as the primary outcome to conclude on what the study was powered for. Evidence mapping does not assess the impact and the risk-of-bias of the included studies; thus, we could not identify research gaps in which a large number of low-impact and poor-quality RCTs exist.

### Implications for research and policy

The small number of RCTs in all types of health care and intervention revealed the limited robust evidence to support decisions in PC. Future studies need to consider these research gaps. Moreover, the COVID-19 pandemic has raised new issues, including teleconsultation, missed care, virtual monitoring, and self-care training for consideration in future research planning.^6,55^ Identified relevant evidence and research gaps through a robust methodological approach may guide evidence-informed health policy; and thus, strengthen the role of PC as a key partner in managing future public health crisis such as the COVID-19 pandemic.^6^ Future RCTs need to go beyond effectiveness, and appropriately address safety and patient-centredness as primary outcomes. Researchers in PC should be reminded that physical, behavioural, and social health are all intertwined, and thus achieving desirable outcomes may require complex interventions targeted not only directly to patients but also to structures and processes of care. This may necessitate the adoption of specific RCT designs, that is, pragmatic, cluster, or stepped-wedged trials. The European General Practice Research Network/World Organization of National Colleges, Academies and Academic Associations of General Practitioners/Family Physicians (EGPRN/WONCA) Europe research strategy could help drive towards that direction.^56^

## Conclusions

Despite the given limitations, we systematically collected a compilation of published RCTs in PC and categorized them according to their primary outcome. We revealed potential research gaps in all types of health care, and in all types of interventions. It should be noted that identified gaps in our study may not necessarily be translated to research needs. However, our work may contribute to determine research needs after considering the importance, desirability, feasibility, and potential impact of identified research gaps, highlighting the necessity of stakeholder engagement in this process.

## Supplementary Material

cmac067_suppl_Supplementary_Figure_S1

cmac067_suppl_Supplementary_Figure_S2

cmac067_suppl_Supplementary_Figure_S3

cmac067_suppl_Supplementary_Table_S1

cmac067_suppl_Supplementary_Table_S2

## Data Availability

The data underlying this article are available in the article and its online supplementary material.
