# Supplementary figures and images for: Health outcomes in primary care: a 20-year evidence map of randomized controlled trials
Source: Fam Pract. 2022 Jul 9;40(1):128–37. doi: 10.1093/fampra/cmac067 (PMC9909671; doi:10.1093/fampra/cmac067)

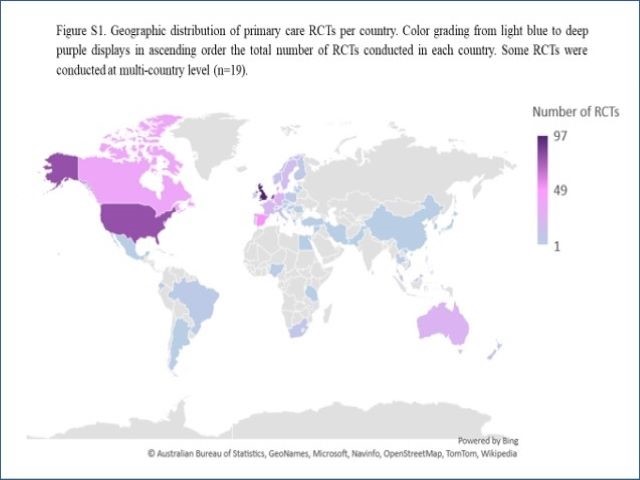

Supplement: cmac067_suppl_Supplementary_Figure_S1 [file cmac067_suppl_supplementary_figure_s1.jpeg]

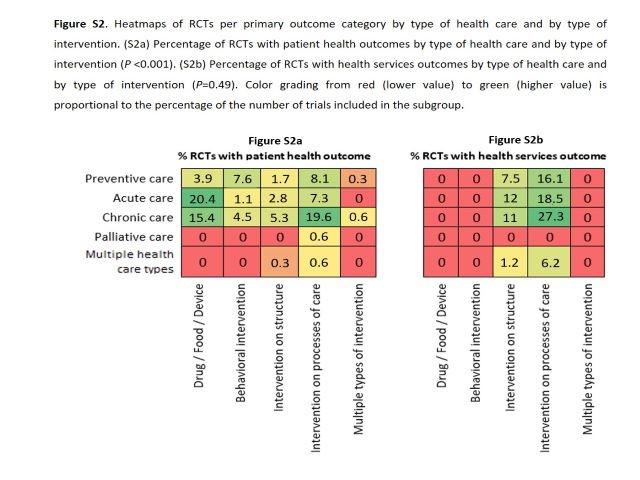

Supplement: cmac067_suppl_Supplementary_Figure_S2 [file cmac067_suppl_supplementary_figure_s2.jpeg]

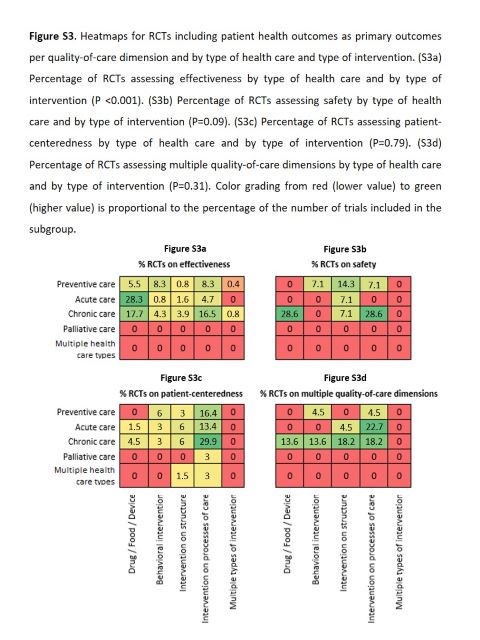

Supplement: cmac067_suppl_Supplementary_Figure_S3 [file cmac067_suppl_supplementary_figure_s3.jpeg]
