# Supplementary material for: Health outcomes in primary care: a 20-year evidence map of randomized controlled trials
Source: Fam Pract. 2022 Jul 9;40(1):128–37. doi: 10.1093/fampra/cmac067 (PMC9909671; doi:10.1093/fampra/cmac067)
Supplement: cmac067_suppl_Supplementary_Table_S2 [file cmac067_suppl_supplementary_table_s2.docx]

**Table S2.** Search algorithms

| *Pubmed search algorithm* (last search on 01/10/2020)  ("Family Practice"[Mesh] OR "Primary Health Care"[Mesh] OR "Physicians, Family"[Mesh] OR “family medicine” OR “family practice” OR “general medicine” OR “general practice” OR “general internal medicine” OR “family physician” OR “general practitioner” OR “primary care” OR “primary health care”) AND ("Clinical Trials as Topic"[Mesh] OR "randomized controlled trial"[pt] OR "controlled clinical trial"[pt] OR randomized[tiab] OR placebo[tiab] OR randomly[tiab] OR trial[tiab]) |
| --- |
| *Scopus search algorithm* (last search on 01/10/2020)  ( ( KEY ( "Family Practice" ) OR KEY ( "Primary Health Care" ) OR KEY ( "Physicians,Family" ) OR ALL ( "family medicine" ) OR ALL ( "family practice" ) OR ALL ( "general medicine" ) OR ALL ( "general practice" ) OR ALL ( "general internal medicine" ) OR ALL ( "family physician" ) OR ALL ( "general practitioner" ) OR ALL ( "primary care" ) OR ALL ( "primary health care" ) ) AND ( KEY ( "Clinical Trial" ) OR KEY ( "randomized controlled trial" ) OR KEY ( "controlled clinical trial" ) OR TITLE-ABS ( randomized ) OR TITLE- ABS ( placebo ) OR TITLE-ABS ( randomly ) OR TITLE-ABS ( trial ) ) AND DOCTYPE ( ar ) AND SRCTYPE ( j ) AND LANGUAGE ( english ) ) AND NOT DBCOLL ( medl ) |
| *CENTRAL search algorithm* (last search on 01/10/2020)  ("Family Practice"[MeSH] OR "Primary Health Care"[MeSH] OR "Physicians, Family"[MeSH] OR "family medicine" or "family practice" or "general medicine" or "general practice" or "general internal medicine" or "family physician" or "general practitioner" or "primary care" or "primary health care":ti,ab,kw) NOT (“animals"[MeSH]) NOT ("PubMed" in Trials) NOT ("Scopus" or "MEDLINE" or "EMBASE":so in Trials) NOT ("conference":so) |
